# Retroversions of the Uterus1Read at the Niagara Falls Medical Society January 4, 1909.

**Published:** 1909-03

**Authors:** Herman E. Hayd

**Affiliations:** Buffalo, N. Y.


### Retroversions of the Uterus.[1]

By HERMAN E. Hayd, M.D., M.R.C.S., Eng., Buffalo, N. Y.

POSTERIOR displacements of the uterus are universally recognised as being capable of producing many different and diverse symptoms, either from pressure upon neighboring organs or reflexly upon remote ones. Modern pathology has taught us the importance of recognising these displacements early, since they predispose to structural changes not only in the uterus and its lining membrane, but, by continuity of tissue, involve the adnexa.

In married and child-bearing women these displacements are very common, and are perhaps responsible for more suffering than often results from the more serious diseases of the tubes and ovaries. They are also of frequent occurrence in the young and unmarried. These deviations are no doubt occasionally associated with a defective development of the organ and conse-

1. Read at the Niagara Falls Medical Society January 4, 1909.

quently some of these women are not favorably influenced by any operation which has for its object the mere placing of the uterus in anteposition. Some are due to injuries and accidents during early girlhood and puberty, but the great majority of them are the result of accidents and complications consequent upon infections, childbirths and abortions.

It is fair to assume that a normal uterus with healthy appendages poised in a healthy body should perform its functions in a painless and natural manner and, other than a sense of discomfort, nothing should mark the menstrual crisis. This Utopia of physical development is not often seen, because varying degrees of pain, from slight distress to the greatest agony, is the unfortunate picture of many girls, due to numerous causes, both constitutional and local. Various forms of dysmenorrhea so graphically described by our writers on diseases of women are no doubt due to a malplaced uterus. Perhaps owing to the obstruction it causes to the menstrual flow on account of the flexion but also to the congestion and associated thickening of the endometrium.

There is a popular belief that so-called womb trouble exists only in married women, or women who have borne children or have had miscarriages or accidents of some kind, and I occasionally meet a medical man who is of the same opinion. As a result, young girls and maiden women are permitted to go on suffering for years and often into chronic invalidism without relief. Irreparable diseases of the tubes and ovaries result, and various radical and mutilating operations become necessary. Medical men have shrunk, and with some justice, from instituting vaginal examinations in young women, feeling that such examinations are often unnecessary—as their causes of ill health are more constitutional than local—and that they not only shock the highly sensitive nature of a modest young girl but di'ect her mind to her genital organs. All this is true, but having once demonstrated that in a particular case the symptoms are either dependent upon or increased and accentuated by a possible posterior uterine displacement, an examination should at once be made by the rectum, and, if such a condition exist, then there is but one course indicated, and that is a curettage and Alexander operation. Since young women can seldom wear pessaries and their displacements are not cured by anything but surgical measures.

In discussing this important question, so far as treatment is concerned, the subject must divide itself immediately into two great classes—retrodisplacements that are complicated, and those that are not complicated—because the treatment involved is definite and scientific as applied to each class, and just as we err in properly interpreting this mode of treatment, so we shall have success or failure. Retrodisplaced uteri are complicated when there exists marked tubal and ovarian disease; when adhesions bind the uterus to adjacent tissues so that easy, free and complete reposition is impossible; when retraction of the broad ligaments has taken place as a result of a previous pelvic cellulitis or retraction from distorted tubes; in fact, anything which interferes with the normal mobility of the uterus would contraindicate all extraperitoneal surgical procedures. On the contrary, a uterus which is perfectly movable, whose normal excursions are not impeded by retraction of tissue or adhesions to neighboring structures, whose tubes and ovaries are healthy—at all events, not much involved, excepting perhaps in a slight catarrhal or congestive process—and where not more than the first degree of prolapse exists, can be said to be the seat of an uncomplicated retrodisplacement, and this condition can often be relieved and occasionally cured by mechanical measures, such as the use of posture, tampons or pessaries; but when these measures fail, or there continued use is objected to, the retrodisplacement can be permanently removed by a simple extraperitoneal operation, and preferably that devised by Alexander and called after him, which consists—as you all know—in shortening the round ligaments extraperitoneally.

So far as the applicability of the Alexander operation is concerned, the displacement is still uncomplicated even though the uterus be the seat of a severe laceration of the cervix with its associated subinvolution, because the Emmet operation or an amputation of the cervix can be depended upon to cure these complications; in fact, the case may be said to be uncomplicated when the uterus is not seriously involved structurally by neoplasms, degenerations or malignancy. If there be relaxation of the perineum and vagina, with cystocele and rectocele, an anterior and posterior colporrhaphy and perineorrhaphy can be performed at the same time by any reasonably rapid operator without materially increasing the dangers of the operation.

The difficulties of diagnosis in enabling the physician to decide whether a given case is complicated or uncomplicated should not under most circumstances be great, and particularly where the patient is examined under an anesthetic. Pelvic exudates can be felt, enlarged tubes and ovaries can be mapped out, particularly pyosalpinx and ovarian abscess with adherent omentum and adjacent viscera. A uterus, when strongly fixed, should without fear or error be definitely diagnosticated; in other words, all gross pathology can usually be made out by any man of ordinary experience. Sometimes great difficulties in the way of diagnosis occur, and the surgeon occasionally sees a case where there exists slight tubal and ovarian involvement or where long thin and fragile adhesions bind the uterus posteriorly, but still permit much mobility of the organ, in fact, even permit it to be thrown into quite a degree of anteversion; or the tubes and ovaries can be perfectly healthy, so far as a macroscopical examination can determine, and yet these long and delicate bands of adhesion can hold the upper and posterior surface of the uterus backwards and yet can be stretched out so as to permit it to come so far forward as to be nearly within normal limits. A careful bimanual examination will, however, determine this rare complication—namely, if the hand upon the abdomen, which is forcing the uterus forward, and the fingers in the vagina, which are elevating and releasing the uterus, be both removed, the organ will at once fall back into its previous acquired retroposition; while if there were no adhesions and bands of retraction the uterus would remain indefinitely forward by the intraäbdominal pressure.

Again, a uterus will sometimes be so firmly wedged into the hollow of the sacrum that it will be impossible to elevate it until the cervix is forcibly pulled downwards with a volsellum forceps, and the organ manipulated in different directions, when the body hitherto markedly tipped can be easily thrown into extreme anteflexion. Such experiences I have had, and it seemed impossible to elevate the uterus, and yet when the abdomen was opened, it came forward without any trouble, and it was found with the tubes and ovaries to be normal and healthy. Fortunately these cases are comparatively infrequent, and represent the border line, where any of us will occasionally err; and perhaps we will open the abdomen—as I have done—when an intraperitoneal operation was not necessary; or we may do an extraperitoneal operation when an abdominal or vaginal section was indicated.

For the treatment of complicated retrodisplacements the abdominal cavity must be opened, and whether this be done by the median incision or by the vaginal route I shall not burden you in this paper, nor with the advantages and the disadvantages of these respective methods of operating, because usually each operator chooses the route he can do best work through, and as a result, perhaps, his number of cures is greatest and his mortality least. Whatever method be chosen the uterus must be released, diseased tubes and ovaries must be removed; or treated conservatively in the hope of saving as much of these structures as is possible and consistent with safety and future well-being. Raw edges must be covered over with peritoneum so as to prevent adhesions between raw surfaces; the omentum must be everywhere released, and all ragged and torn portions must be ablated and the bleeding ends carefully tied off.

Pockets of pus must be opened and cleaned out, and perhaps drainage provided for by tube or wick passed anteriorly through the abdominal wound or through a vaginal slit or opening, and a clean and perfect toilette of the peritoneal cavity be made as quickly as possible. Various methods and devices are employed to keep the released uterus forward. Some surgeons sew the uterus to the abdominal incision, taking in the peritoneum or muscle or fascia, or all three structures in the bite of the suture, and doing what is ordinarily called a ventral fixation and ventral suspension. Others loop up the ligaments and sew the opposed peritoneal surfaces together with silk or chromicised catgut, previously irritating the parts which will touch with 1 to 2,000 corrosive sublimate solution, or gently scarifying the surfaces so as to destroy the smooth endothelial covering. Others loop together the two ligaments in front of the uterus, or sew the loop of each ligament forward by bringing it about one inch from the abdominal incision up through a hole in the rectus muscle, and then fixing it by suture—the so-called Gilliam operation.

All of these measures are reasonably successful, but failures occur and the uterus occasionally falls back, either by the ligaments stretching, or because the adhesions were not strong enough to stand the violent muscular efforts which these parts are subjected to. In a general way it may be stated that a uterus with a healthy tube and ovary should not be fixed forward during the child-bearing period, and if possible, such a uterus when retrodisplaced should be pulled forward by some of these intraperitoneal methods of shortening the round ligaments. Sometimes the uterosacral ligaments can be advantageously shortened, because when they are taut, they tend to pull the cervix upwards and backwards, and thus help to keep up a normal anteposition. If, however, there be marked prolapse, it may be necessary to fixate the organ forward, so as to help to support the vaginal structures below.

Certain operations are performed upon the utero vesical ligaments through the vagina, but I have no experience with that method of correcting retrodisplacements. Another method of treating complicated retrodisplacements is to open the cul de sac and break down any adhesions which exist, place the uterus well forward, and then pack through the opening gauze to hold the organ in place, and to make new adhesions, so that the anteposition shall remain permanent. I have never given this method any consideration, as I am satisfied it is a dangerous procedure and too uncertain in its results; first of all, it is impossible to limit any adhesive inflammation, and secondly, there is no guarantee that these new adhesions will not retract and extend to other adjacent structures and do more harm than good.

Can retrodeviations of the uterus exist without producing any symptoms? I should answer yes, but rarely, and usually sooner or later, a definite train of symptoms will be associated with every retrodisplacement. In fact, I know of no condition capable of masquerading itself in so many different kinds of symptoms and expressions as a retrodisplaced uterus; for not only will there be local symptoms, such as pain in the back and bearing down pains, leucorrheal discharge, increased or diminished menstrual flow, with or without pains, but all kinds of reflex nervous symptoms—headaches, eye troubles, stomach distress and dyspepsia, intestinal indigestion with the allied toxemias, bladder irritation, palpitation of the heart, neurasthenia, hysteria, and all their unhappy and varied symptomatology.

Can a retrodisplacement exist for any length of time and not produce serious tubal and ovarian involvement? Certainly, and I know women, and you have them under your care, who have had retrodisplacements for years and bear children. After each confinement the uterus falls back, and yet the tubes and ovaries remain functionally and structurally well, and I have operated on scores of women who have had a uterine retrodisplacement for years, and who were cured by operative measures and have subsequently borne children, and the uterus and tubes and ovaries have remained in normal condition. However, such uteri are apt sooner or later to be complicated, and for that reason, demand treatment. In some women, the treatment should be energetic, while in others, it may be tentative and watchful. I am certain there exists a large class of suffering women, invalided more or less as a result of uncomplicated uterine displacements, and it is for that class of sufferers I wish to present the results of my experience, and particularly with the Alexander operation, which I have been a constant and continued advocate of in original papers and discussions before medical societies for the past ten years.

In the November, 1904, number of the *American Journal of Obstetrics and Diseases of Women and Children*, is published a paper which I read in St. Louis at the meeting of the American Association of Obstetricians and Gynecologists, in which I detailed the results of my experience with this operation, supplemented with various plastic operations upon the cervix, vagina and perineum, in twelve women, who subsequently became pregnant, and in every one the uterus stood the test of pregnancy and parturition—to one, three children were born; to others, two. In fact, in every case of pregnancy which has followed the Alexander operation in my hands the uterus has remained in position, and I believe that the pregnancy contributed largely to the success of these operations and the general well-being and comfort of these women. A new uterus was made, muscular tissue in the round ligaments was increased in amount, adhesions to the scar and skin were separated and torn loose as the growing uterus went higher into the abdominal cavity, and after the labor had taken place, a normal involution of all these structures set in, and a natural and satisfactory and happy puerperium resulted.

I have no reason to doubt that many other women upon whom I have done the Alexander operation have also borne children, but these cases I personally confined, or they were confined by my intimate friends, so that I had the opportunity of examining the women repeatedly since their babies came.

Since the above paper was written three or four of these women have borne more children and the uterus is still in perfect anteposition.

I have never failed to find the ligaments, and I am satisfied they are always present; sometimes, however, so small that unless one is skilled in the technic of the operation they would be lost or irreparably broken. The operation as done by me is to pick up the terminal ends of the expanded and spread-out ligament at its origin at the external ring, gradually and carefully pull them together, so as to get a better hold of the stronger and bigger part of the ligament in the canal, and carefully pull it out three or four inches, as the case requires. This traction must be always directed inwards towards the spine of the pubis, or the ligament might be broken if it were pulled outwards against the hard borders of the ring, and it must be strong enough and continued long enough until the body of the uterus can be felt against the abdominal wall; then cut off the frayed and bruised part of the ligaments and fix the stumps to the pillars of the ring. The canal is never opened deliberately, and is only nicked or slightly opened when the ligament does not pull out easily, and the internal ring and the abdominal cavity are never opened under any pretense whatsoever. Failures have occasionally occurred; in my first fifty operations more often than in my next 100 cases, due to inexperience; and I believe, although it is impossible for me to follow all of my cases, as many of them are in the tenement houses of Buffalo, my percentage of returns of the backward displacement is not 5 per cent.

If a thin ligament is met, more care must be taken in the traction, and a support must be worn for some months after the operation. Often the ligament at its pubic end will be as thin as the nerve; in fact one may be in doubt whether it be the nerve we are dealing with, but if it be carefully nursed and followed up into the canal, all difficulty will be removed. And that same ligament, often so thinned out at its origin at the pubis, when pulled out farther will be found to be a good, wholesome band and capable of enduring any kind of strain when properly and scientifically dealt with.

In some of my early cases I did not use a pessary after the operation, and the displacement recurred; so I now make it a rule to employ a pessary in nearly every case for two, three or four months after the operation, and I see the patients occasionally afterwards if possible, so as to be certain that the ligaments will stand the work imposed upon them after the support is removed. In two unmarried virgin women the uterus fell back, and after a support was worn some months retraction of the ligaments took place, and the uterus has remained in perfect anteposition for years. In other words, a ligament which has stretched out after operation, will often retract as its tissues are young and elastic. Also in two of my cases where pregnancy took place, in one, after the third child, the uterus fell back, and in the other after the fourth child and yet after wearing a support for a few months the uterus remained in beautiful anteposition.

Suppuration occurred frequently in my early operations, but with a better technic and the use of gloves it is seldom or never seen. Catgut and kangaroo tendon are used in all the operations, the former for ligating vessels and bringing the skin together, and the latter for sewing the cut ends of the ligaments to the pillars of the ring, or closing the canal, if it has been opened for any reason, such as a previous complicating single or double hernia. Sometimes a painful scar remains, because the perhaps engaged in the bite of the suture. This can be avoided by a little patience and forethought, but when it exists, it usually is not a permanent affliction, and passes away in a few weeks or months. Sometimes the nerve is torn in the manipulations, but other than a slight anesthesia in the parts for a few months is not a matter of serious inconvenience.

Hernia has never occurred but once in my work, and I see no reason why it should if the canal is not opened, but if from any cause the canal or a portion of it must be opened, if care is taken to properly and carefully bring the edges together, perfect union should take place. The woman should be kept in bed for three weeks after the Alexander operation, and a pessary should be inserted into the vagina after the curettage, or after the operation has been completed, or as soon as the perineum and vagina will permit, if plastic operations have been done at the same time.

In the treatment of old cases of retroflexion greater difficulties are met with than in similar cases of retroversion, because tissue changes have taken place in the uterus at the point of flexion; the anterior wall has been permanently stretched and the posterior is kinked and hardened, and it is often impossible to keep such a uterus forward simply by traction on the round ligaments. I have in rebellious cases, after a thorough and extremely free dilatation of the cervix, with subsequent curettage, put into the uterus a bent intrauterine stem with a slot on the side for drainage, and I have made the patient wear this for some weeks. It keeps up a continuous and persistent dilatation, and helps the absorption of adventitious tissue. I am satisfied this is a very important matter, because a uterus which has much rigidity left at the point of flexion pulls back, and soon the intra-abdominal pressure is directed on the top and front of the fundus, instead of posteriorly, and thus one of the most important forces in the production and development of posterior displacements is again at work. A stem pessary must always be watched, but when the patient is in bed and prepared most carefully for operation, there is not much danger of infection, and therefore this much-dreaded instrument can be employed with comparative safety, and coupled with the Alexander operation, it accomplishes much even in this difficult class of cases.

Let me now conclude this paper by making these deductions:

1. A plea for the more careful examination of young women by competent and skilled men who can undertake any operative measures that are necessary.

2. Every case of retrodisplaced uterus in the young or unmarried or married woman may not require any treatment.

3. If they produce a definite symptomatology, the Alexander operation should be employed, if the case be an operable one; that is, if the uterus is freely movable and the tubes and ovaries are healthy.

4. Retroversions and retroflexions in the young and unmarried should never be treated by pessaries, but by the Alexander operation. Tampons and pessaries have their place in retro-displacements in married women or women who have been pregnant, but they accomplish practically nothing in the displacements of young women.

5. The Alexander operation is safe and without mortality incident to the operation, and no harm can come from its proper performance; even if the uterus subsequently falls, the patient is no worse off than she was previous to the operation.

6. It does not in any way interfere with pregnancy and future child-bearing, but on the contrary materially helps the possibility of pregnancy.

7. No pain or distress follows the operation if the case operated upon be properly selected; and if pain and suffering result, there existed at the time of the operation latent tubal and ovarian trouble, which sooner or later perhaps would have required a radical operation. If it becomes necessary to do a celiotomy on a person who previously had an Alexander operation, the uterus will be found in its normal anteflexed position, which is necessary in every case, whether the tubes and ovaries are removed or not, to insure good health and freedom from future suffering.

8. Complicated retroversions must be treated on general surgical principles and preferably by the abdominal route where the benefit of sight enables us to not only do conservative surgery, but the very best surgery.

493 DELAWARE AVENUE.

